# Mapping the Small RNA Content of Simian Immunodeficiency Virions (SIV)

**DOI:** 10.1371/journal.pone.0075063

**Published:** 2013-09-23

**Authors:** Markus Brameier, Wiebke Ibing, Katharina Höfer, Judith Montag, Christiane Stahl-Hennig, Dirk Motzkus

**Affiliations:** 1 Primate Genetics Laboratory, German Primate Center, Göttingen, Germany; 2 Unit of Infection Models, German Primate Center, Göttingen, Germany; French National Center for Scientific Research - Institut de biologie moléculaire et cellulaire, France

## Abstract

Recent evidence indicates that regulatory small non-coding RNAs are not only components of eukaryotic cells and vesicles, but also reside within a number of different viruses including retroviral particles. Using ultra-deep sequencing we have comprehensively analyzed the content of simian immunodeficiency virions (SIV), which were compared to mock-control preparations. Our analysis revealed that more than 428,000 sequence reads matched the SIV _mac_239 genome sequence. Among these we could identify 12 virus-derived small RNAs (vsRNAs) that were highly abundant. Beside known retrovirus-enriched small RNAs, like 7SL-RNA, tRNA^Lys3^ and tRNA^Lys^ isoacceptors, we also identified defined fragments derived from small ILF3/NF90-associated RNA snaR-A14, that were enriched more than 50 fold in SIV. We also found evidence that small nucleolar RNAs U2 and U12 were underrepresented in the SIV preparation, indicating that the relative number or the content of co-isolated exosomes was changed upon infection. Our comprehensive atlas of SIV-incorporated small RNAs provides a refined picture of the composition of retrovirions, which gives novel insights into viral packaging.

## Introduction

Within the last decade, a diverse class of non-coding RNA molecules (ncRNAs) was found to orchestrate a large number of physiological processes. The most prominent population of ncRNAs was referred to as microRNAs (miRNAs) that induce gene silencing by translational repression or mRNA decay [1]. Derived from primary transcripts, biogenesis of miRNAs starts with a precursor molecule (pri-miRNAs) that is processed by the nuclear RNase III enzyme Drosha. After nuclear export, the pre-miRNA is cleaved by the cytoplasmic RNase III enzyme Dicer that produces the mature miRNA of around 22 nt in length. To exert their function, miRNAs are incorporated into the RNA-induced silencing complex (RISC). Complementary binding of the miRNAs to its respective target transcripts then leads to mRNA degradation or translational repression, influencing physiological or promoting pathological processes. MiRNAs have been found in virtually all eukaryotic organisms. An increasing number of studies showed that miRNAs are also encoded and expressed by a variety of DNA viruses, including several members of the Herpesviridae and Polyomaviridae families (for review, see [2]). Furthermore, deep-sequencing approaches lead to the identification of short, viral-derived sequences (vsRNAs) in RNA viruses. To date, the function of these fragments in Hepatitis C-, Vesicular Stomatitis-, West Nile-, Dengue-, and Polio-virus infected cells remains unknown [3].

In addition, retroviruses, such as the human immunodeficiency virus (HIV-1), have been reported to encode miRNAs [4-7] and vsRNAs [8,9]. Retroviruses are RNA viruses that integrate in the host genome via a proviral DNA intermediate. The primary genomic RNA (gRNA) serves as mRNA and via splicing produces many subgenomic mRNAs. During the maturation of retroviral particles, a proportion of the full length RNA is packaged into progeny virions as a dimer of two identical copies. Selective packaging of this gRNA depends on the RNA-encoded Psi (Ψ) region that is recognized by the nucleocapsid domain of the structural protein Gag [10,11]. The Gag polypeptide also binds to several cellular RNAs [12] that are selectively packaged into the virion. In HIV-1, the majority of these cellular RNAs belongs to the family of RNA polymerase III (Pol III) transcribed ncRNAs, such as tRNA^Lys^ and tRNA^Lys^ isoacceptors. Here, tRNA^Lys3^ anneals to the primer binding site (PBS) of the gRNA, where it serves as initiator of reverse transcription [13,14]. In addition, 7SL RNA, which is one component of the signal recognition particle (SRP) ribonucleoprotein complex, is highly enriched in HIV-1 derived virions [15]. Using mutated HIV-1 particles, there is evidence that the nucleocapsid domain of Gag mainly contributes to the selective packaging of Pol III transcribed cellular small RNAs into HIV-1 virions [16]. Recent evidence indicated that 7SL RNA could also be retained in the virion as endoribonucleolytic fragment [17]. To date, only the above mentioned cellular RNAs and RNA fragments have been identified within the virion, whereas HIV-1 encoded vsRNAs were solely detected in HIV-1 infected cells [8,18].

In this study we examined RNA packaging of the simian immunodeficiency virus (SIV _mac_239) using ultra-deep sequencing technology. Our data confirm incorporation of several known non-coding RNAs, such as 7SL RNA and tRNA^Lys^, but also provide evidence for the residence of other host cell-derived small RNAs in the virions, that have not been described yet. In addition, we detected several vsRNAs that originated from SIV. The results provide a first atlas of a retroviral small virionome that essentially contributes to our understanding of virion composition.

## Materials and Methods

### Infection of C8166 cells with SIV _mac_239

The human T-cell line C8166 (NIH AIDS reagent 404 [19]) was cultured in RPMI-medium supplemented with stabilized L-Glutamine (Life Technologies), 10% fetal calf serum (FCS, Pan Biotech), and Pen/Strep at 37°C in 5% CO_2_ and 95% humidity. Cells were passaged three times per week and kept at a density between 5 x 10^5^-4 x 10^6^ cells/ml in 75 cm^2^ or 175 cm^2^ cell culture flasks (Sarstedt). Cells were infected with simian immunodeficiency virus (SIV _mac_239, Acc. no. M33262) prepared from previously infected C8166 cells at an MOI of 0.01. 1 x 10^8^ cells were suspended in 48 ml fresh medium plus 2 ml of the SIV virus stock. SIV was allowed to adsorb to the cells at 37°C for 1.5 h, then cells were washed with prewarmed PBS and resuspended in new flasks at 5 x 10^5^ cells/ml. Medium was replenished every 2-3 days and the density was kept as indicated above until day 9 post infection (dpi). Virion-containing cell culture supernatants were pooled from 7 dpi and 9 dpi. As a control, supernatants derived from non-infected cells were cultured in parallel and processed in the same way.

### Enrichment of SIV virions

Virion-containing and non-containing cell culture supernatants were centrifuged at 1,800 x g for 15 min at RT. The supernatants were again cleared by an additional centrifugation step at 16,000 x g at 4°C for 60 min. In order to precipitate virions, 0.3 M NaCl and 10% (w/v) polyethylene glycol 6000 (PEG, Roth) were added to supernatants. The mixture was incubated for 2 h at 4°C on a roller device. Precipitates were sedimented at 4,500 x g at 4°C for at least 8 h. The resulting PEG-pellets were resuspended in DNase/RNase buffer (100 mM Tris/HCl, 25 mM MgCl_2_ 5 mM CaCl_2_, pH 7.5) containing 5 U/ml DNase I (Thermo Scientific), 350 µg/ml RNase A (Qiagen) and 100 U/ml RNase I (Ambion) and incubated for 4 h at 37°C. The reaction was stopped by the addition of Trizol (Life Technologies). The sample that was processed from SIV-infected C8166 cell culture supernatants will be referred to as „SIV-sample“, the corresponding sample derived from non-infected cell culture supernatant as „mock sample“. Total RNA from the SIV- and the mock sample was isolated according to the supplier’s instructions (Trizol RNA isolation protocol, Life Technologies).

### Characterization of PEG-isolated virions

Alternatively, the SIV virion containing PEG-pellet was resuspended in DNase/RNase buffer alone or with DNase/RNase and incubated for 4 h at 37°C. From the resulting samples RNA was isolated using a NucleoSpin RNA virus kit (Macherey & Nagel). Infectivity of isolated virions was quantified by a limiting dilutions assay using C8166 cells as previously described [20,21].

### Determination of SIV copy number by QPCR

Resuspended PEG pellets derived from SIV- and mock-infected cells were used to enrich viral RNA using the QIAamp viral RNA Mini Kit (Qiagen) according to the supplier’s instructions. The eluate was precipitated with 2.5 (v/v) ethanol and 1/10 (v/v) 3 M sodium acetate, pH 5.2, washed twice with 70% ethanol, dried, and resuspended in 10 µl H_2_O. From this preparation 1 µl was reverse transcribed with random hexamer primers (Thermo Scientific) using Bioscript reverse transcriptase (Bioline) in the presence of 10 U RNase inhibitor (RiboLock, Thermo Scientific) according to the supplier’s instructions. Quantification of *gag* copies was performed as previously described [22] with some modifications. In brief, 1 out of 20 µl cDNA and 400 µM SIV *gag*-specific primers (*gag*-for: ACCCAGTACAACAAATAGGTGGTAACT; *gag*-rev: TCAATTTTACCAGGCATTTAATG) were diluted in 1x PowerSybrGreen Mix (Applied Biosystems) to a final volume of 25 µl. For the generation of standard curves a plasmid encoding for SIV *gag* was diluted from 10^8^ to 10^3^ copies/µl and used instead of cDNA. After initial denaturation at 95°C for 10 min, QPCR was performed in duplicates for 45 cycles with 45 sec annealing at 58°C and 15 sec denaturation at 95°C. Amplification, data acquisition and analysis were performed using the ABI Prism 7700 sequence detection system (Applied Biosystems).

### Ultra-deep sequencing of small RNAs

Samples were sequenced on an Illumina HiSEQ 2000 device by a commercial service (Fasteris SA, Switzerland). In brief, 10 µg total RNA from SIV- and mock cell culture supernatant, respectively, was used to isolate small RNA in the range of 20-30 nt by acrylamide gel purification. The small RNA was decapped with Tobacco Acid Pyrophosphatase (TAP) treatment, separately ligated to identical bar-coding 5‘- and 3‘-adaptors, acrylamide gel purified, reverse transcribed, and PCR-amplified. Libraries were sequenced in parallel on two different flow cells. Sequence data were trimmed to retrieve the original small RNA inserts.

### Bioinformatic analysis

The Illumina read libraries were converted into FASTA file format and further processed using custom Perl scripts. First, read files were compressed by compiling doubles, i.e. perfectly identical read sequences were encoded with a frequency tag. The obtained non-redundant libraries were filtered for reads that were (I) >15 nt in length and/or (II) occurred more than 10 times as a cut-off for reliable sequence reads. Both compression and filtering significantly reduced the time and overhead of subsequent analyses by a factor of ~150. For identification and annotation we applied NCBI BLAST (blastall) version 2.2.18 with options -p blastn -e 1000 -v 10000 -b 10000 -F F -m8 and default settings otherwise. Annotated expression profiles were derived from tabular BLAST output for miRNAs (based on the miRBase database, release 19.0) and a selected set of other small ncRNAs (see Results). If not stated otherwise, only reads with 100% identity over the full read length minus a tolerance offset of not more than 2 nt (if the overall matching length did not drop below 15 nt) were selected.

### Data deposition

Data were deposited at the European Nucleotide Archive and can be accessed at http://www.ebi.ac.uk/ena/data/view/PRJEB4152.

## Results

### Experimental setup for small RNA identification in SIV virions

Packaging of small RNAs into virions is a common feature known from DNA [23-26] and RNA viruses [17,27]. During the assembly of HIV-1 viral particles several tRNAs [13,28] and other non-protein coding RNAs like 7SL RNA [15] are non-randomly incorporated into progeny virions. To determine whether other small RNAs are also packaged into retroviral particles we analysed the small RNA content of simian immunodeficiency virions (SIV) by ultra-deep sequencing. Because of the superior yield, the human T-cell line C8166 was infected with SIV _mac_239 with an MOI of 0.01 and cultivated for seven to nine days. SIV virions were enriched by consecutive centrifugation and final PEG precipitation steps (see Material and Methods). The used strategy resulted in a high recovery rate of virions, but due to the expected co-purification of host cell derived exosomes, the virions were obtained in a limited purity (see discussion). To overcome this limitation we used control supernatants derived from mock-infected cells that were processed identically and in parallel throughout our experiment. In detail, cell culture supernatants from SIV-infected and mock-infected cells were cleared from cell debris by low-speed centrifugation. Next we sedimented microvesicles and apoptotic bodies by centrifugation at 16,000 x g for 60 min according to a published protocol [29]. The supernatant was treated with PEG, which presumably resulted in sedimentation of exosomes in the control supernatant and exosomes plus virions in the SIV-containing supernatant, respectively. The derived pellets were resuspended in buffer and treated with high amounts of ribo- and desoxyribonucleases to degrade non-vesicular and non-virion protected DNA and RNA.

To estimate whether the treatment with DNase/RNase affected encapsidated viral nucleic acids, we analyzed both, the copy number of viral RNA and the infectivity of virions prior to, after, and by omission of the DNase/RNase treatment step. Aliquots from the PEG-pellet, mock-treated and nuclease treated samples were used to isolate viral RNA. QPCR analysis showed that the copy number of virus was not significantly different between the three samples (Figure S1). In addition, isolated virions were tested for infectivity by a limiting dilution assay and subsequent calculation of the present titer. In line with the QPCR data, the three samples did not show marked differences in the amount of infectious SIV, supporting that the nuclease treatment did not influence the integrity of the virions.

Subsequently, apparently lipid-layer protected nucleic acids residing in virions and/or exosomes were isolated using Trizol reagent. Spectrophotometric measurement of the isolated RNA derived from 50 ml cell culture supernatant from SIV-infected cells revealed a total amount of 13.3 µg nucleic acid content according to the optical density at 260 nm. In comparison, the yield in mock-infected cells was considerably lower (3.5 µg). The enrichment of viral particles was confirmed by cDNA synthesis and subsequent quantitative real-time PCR (QPCR) for SIV *gag*. Using this quantification method we calculated that the Trizol-isolated PEG-pellet contained approximately 7.5 x 10^9^ SIV RNA copies. According to the genome size of 10535 bases of SIV _mac_239 this amount arithmetically corresponds to 37.8 ng of RNA. Although both, the quantification of RNA by QPCR and photometric quantification of nucleic acids are biased by numerous factors, it is noteworthy that the content of viral genomic RNA only corresponds to approximately 1% of the isolated amount of RNA in the SIV-sample, suggesting that most of the isolated nucleic acids were not SIV-encoding gRNA.

Subsequently, we used ultra-deep sequencing to identify and compare small individual RNA sequences in our preparation. Both, the SIV-infected and mock-treated samples were processed in parallel. After purification of small RNAs with a size range of 20-30 nt, both samples were treated with Tobacco Acid Pyrophosphatase (TAP) and barcoded by the ligation of identical adaptors. The libraries were sequenced in parallel using different flow cells on an Illumina HiSEQ2000 device in single reads. The derived FASTA dataset was trimmed and the sample-derived insertions were compiled according to their frequency (also see Materials and Methods). To exclude rare sequences, individual sequence reads that were detected less than 10 times were excluded from the dataset. Using this cut off we retrieved 1,129,344 sequence reads from SIV-infected cell culture supernatant and 385,191 sequence reads from the mock-treated cells, that were in the range of 15 to 44 nt.

### Identification of vsRNAs in SIV-virion preparation

Since virus-encoded small RNA populations can be incorporated into virions [26] we first aimed to identify SIV-derived small RNAs (vsRNAs). BLAST analysis revealed more than 428,000 sequence reads in the SIV virion sample that matched the SIV _mac_239 genome sequence (Acc. no. M33262). It should be noted that our BLAST filter was set to 100% identity, and that with this setting no matches were found in the mock sample. In addition, none of the sequences were in antisense orientation, thus they must have been originated from the (+)-strand of SIV.

**Figure 1:**
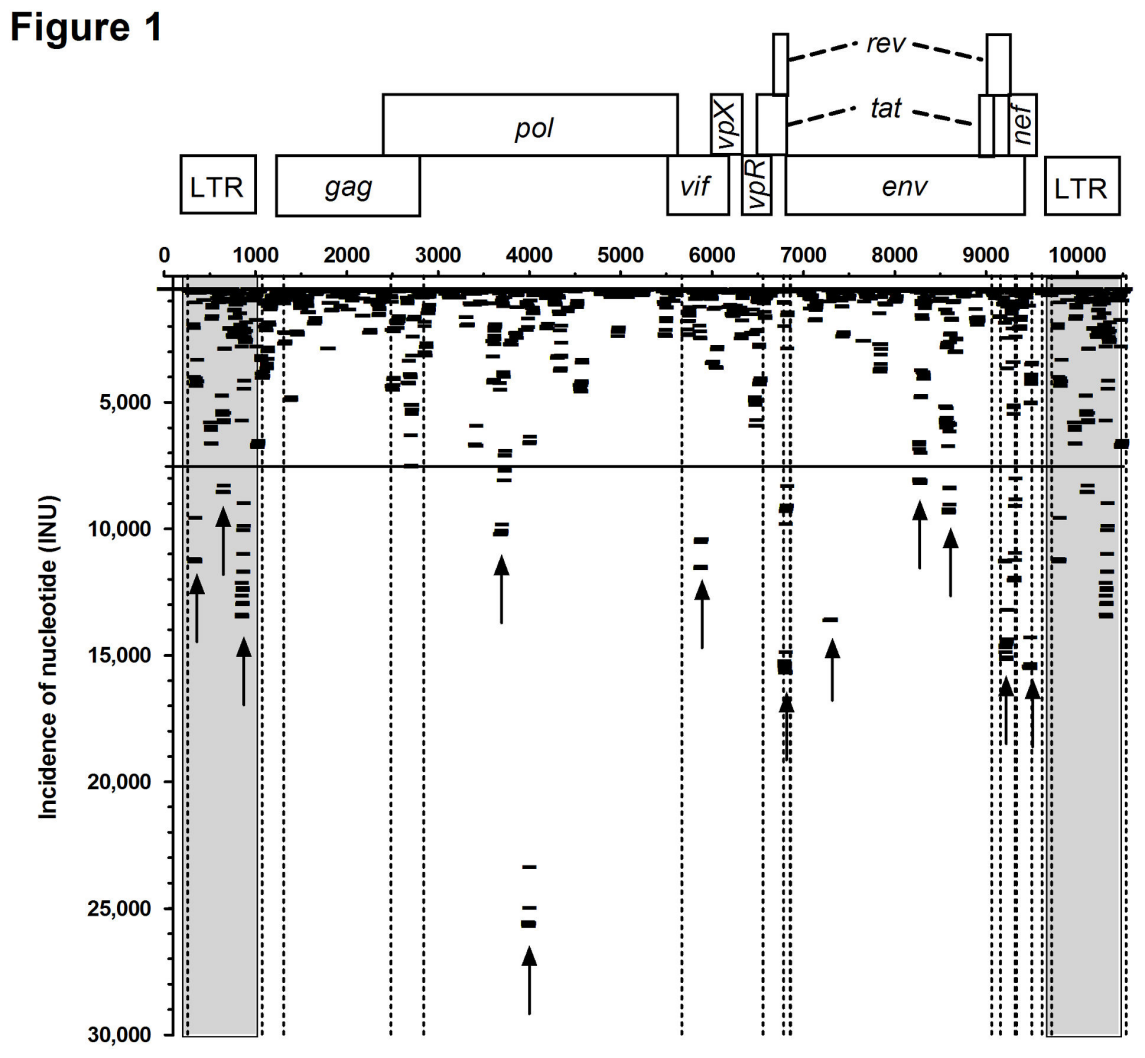
Identification of SIV-derived vsRNAs by ultra-deep sequencing. Incidence of nucleotides (INU) derived from 428,447 individual reads from Illumina HiSeq2000 sequencing were plotted along their position on the SIV _mac_239 genome (Acc. no. M33262.1). Genes encoded by SIV are depicted on top. Dotted lines indicate the start and end positions of the individual exons and coding regions. Shaded boxes indicate identical sequences in the long terminal repeat (LTR) regions. An arbitrary threshold is marked by a horizontal line at 7,500 INU. Arrows indicate 12 regions with highly abundant vsRNAs.

**Figure 2:**
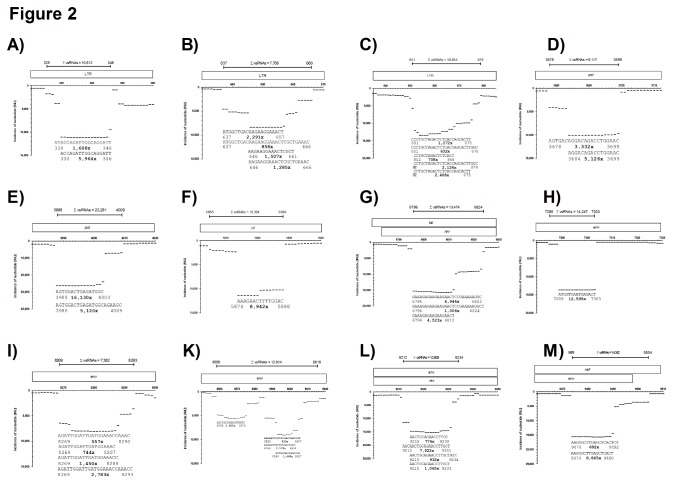
A-M: High resolution maps of individual vsRNAs. High resolution maps of individual regions with INUs > 7,500 depicted from Figure 1. The position of each nucleotide in SIV _mac_239 is shown on the X-axis. Genes encoded by SIV are given on top. The most abundant sequence reads are plotted against the X-axis together with their corresponding 5‘- and 3’-ends. The number of the individual sequence reads is shown in bold. The total number of reads mapping to the respective region is shown on top.

For visualization of the results we counted the incidence of each nucleotide that matched the SIV genome, irrespective of the individual fragment length it was derived from. The incidence of each nucleotide (INU) was then plotted as a function of its position along the retroviral genome. For example, when 100 sequence reads matched SIV genome position 1-25 and 50 sequence reads matched position 5-25, then the INU for position 1-5 is 100, while for position 5-25 the INU is 150. Using this method we mapped the relative distribution of 8.07 million matching nucleotides along the 10,535 nt long coding sequence of SIV _mac_239 (Figure **1**). Our analysis revealed that 7,262 nt out of 10,535 nt of SIV were sequenced 10 times or more. Due to the treatment of our samples with high amounts of RNases prior to isolation of viral RNA, we assumed that some of these sequences could have been generated artificially during sample processing. Indeed, a high proportion of the SIV genome was covered by virtually random fragments (Figure **S2**), presumably resulting from RNA hydrolysis of SIV-derived gRNA. However, some fragments were significantly more abundant than others. For example, in the coding region of *pol* p31 integrase, we did not find any fragment that aligned to region 4,587-4,729 nt, whereas in the upstream sequence 4,444-4,586 nt with comparable GC content 4,870 sequence reads matched the SIV genome. We therefore assumed that these fragments had not been generated by the experimental procedure. In accordance with the definition of Parameswaran and colleagues [3] we will refer to these highly abundant sequences as SIV-derived small RNAs (“vsRNA”) without any *a priori* functional implication. Using an arbitrary threshold of 7,500 INUs, we still detected 12 highly abundant vsRNAs (see arrows in Figure **1**) that were located in the long terminal repeat (LTR), *pol*, *vif*, *tat/rev*, *env* and *nef* region, respectively. Interestingly, some of these vsRNAs are represented by a discontinuous set of overlapping sequence reads, while others are represented almost exclusively by one single sequence (Figure **2**). Several publications showed that miRNAs can vary at their 3’-ends, which typically affects the last 1-3 nucleotides. It has been suggested that this heterogeneity might be due to inaccurate processing by Dicer [30,31], but also alternative explanations have been proposed [32]. Using the INU profiling method, it can be visualized that the vsRNA sequence at nt 9474-9490 (Figure **2M**) is represented by one highly abundant form (sequenced 8,865 times), and a less abundant form with a protruding 3’-end of two nucleotides, thus resembling a DICER-processed miRNA. Furthermore, the vsRNA shows a sharply bounded 5’-end, which is also a hallmark of miRNAs. In contrast, other vsRNA sequence reads matching the LTR at nt 637 to 666 (Figure **2B**) vary extensively at the 5’- and 3’-ends, making it difficult to annotate a single vsRNA sequence. All other vsRNAs were either similar to the former (Figure **2E, F, G, H**), the latter (Figure **2C, I, K, L**), or show a defined 3’-end but a varying 5’-end (Figure **2A, D**). In total, the 12 vsRNAs are covered by approximately 150,000 individual reads, i.e. more than 1/3 of all sequences matching SIV _mac_239, indicating that they were not produced randomly.

The homologue region of HIV-1 TAR-derived miRNA [4] corresponds to 782-834 in SIV _mac_239, which is predicted to fold in a miRNA-like loop structure. In this region, the overall INU was 250, and only position 830-834 reached the cut-off. We therefore assumed that the homologue of the HIV-1-encoded miRNA was not present or not detectable in the SIV virion particles.

Next, we aimed to determine whether the SIV vsRNAs could be derived from hairpin loops that were potentially processed by the cellular RNAi machinery. Thus we used RNAfold [33] to predict respective secondary structures. The vsRNA sequences were set into their up- and downstream context to test whether the most abundant sequence read could be a -5p or -3p miRNA derived from a pre-miRNA precursor. However, we did not find evidence for the existence of a stable hairpin loop for any of the vsRNAs, indicative of a DICER-processed miRNA. Thus, we concluded that the highly abundant vsRNAs were most probably not processed by the known miRNA machinery.

### Low abundance of miRNAs in SIV virion and mock preparations

In our next step we analysed small RNA sequences that did not match SIV. BLAST analysis revealed 8,387 and 6,606 sequence reads in the SIV and mock sample, respectively, which were identical to known human miRNAs (Table **S1**). Interestingly, some miRNAs were highly overrepresented in the SIV sample (Table **1a**), while others appeared to be more abundant in the mock sample (Table **1b**). It should be noted that the number of sequence reads for some of the miRNAs were considerably low, thus the corresponding ratio tends to be overestimated. The highest ratio (37 fold) in the SIV sample was found for hsa-miR-10b-5p. This miRNA has been described to be counteracted by the apolipoprotein B mRNA-editing enzyme catalytic polypeptide-like 3G (APOBEC3G) [34], a protein that is encapsidated in HIV-1 virions [35]. Another prominent candidate appeared to be hsa-miR-3676-5p that showed a 16 fold higher abundance in the SIV preparation. BLAST search of the miRBase-annotated sequence revealed 100% identity to human tRNA^Thr^, indicating that miR-3676-5p might not be a functional miRNA.

**Table 1:** Abundance of exosomal or virion-associated miRNAs.

	**a**)** >twofold higher abundance in SIV**						**b**)** >twofold higher abundance in mock**					
		**SIV**	**mock**	**ratio**				**mock**	**SIV**	**ratio**
	hsa-miR-10b-5p	74	2	**37,0**			hsa-miR-486-5p	12	2	**6,0**
	hsa-miR-3676-5p	866	54	**16,0**			hsa-miR-146a-5p	14	3	**4,7**
	hsa-miR-219-2-3p	12	1	**12,0**			hsa-miR-25-3p	10	3	**3,3**
	hsa-miR-1	112	12	**9,3**			hsa-miR-99a-3p	3	1	**3,0**
	hsa-miR-135a-5p	30	4	**7,5**			hsa-miR-708-5p	3	1	**3,0**
	hsa-miR-181b-5p	21	3	**7,0**			hsa-miR-4508	6	2	**3,0**
	hsa-miR-218-5p	7	1	**7,0**			hsa-miR-186-5p	8	3	**2,7**
	hsa-miR-206	6	1	**6,0**			hsa-miR-199a-3p	18	7	**2,6**
	hsa-miR-335-5p	6	1	**6,0**			hsa-miR-199b-3p	18	7	**2,6**
	hsa-miR-23a-3p	5	1	**5,0**			hsa-miR-451a	20	9	**2,2**
	hsa-miR-181a-5p	45	10	**4,5**						
	hsa-miR-93-5p	4	1	**4,0**						
	hsa-miR-4532	12	3	**4,0**						
	hsa-miR-342-3p	7	2	**3,5**						
	hsa-let-7i-5p	116	36	**3,2**						
	hsa-miR-9-5p	558	181	**3,1**						
	hsa-miR-9-3p	46	15	**3,1**						
	hsa-miR-92b-3p	3	1	**3,0**						
	hsa-miR-3529-3p	13	5	**2,6**						
	hsa-let-7e-5p	25	10	**2,5**						
	hsa-miR-98-5p	5	2	**2,5**						
	hsa-miR-19a-3p	22	9	**2,4**						
	hsa-miR-137	12	5	**2,4**						
	hsa-miR-136-5p	12	5	**2,4**						
	hsa-miR-100-5p	165	70	**2,4**						
	hsa-miR-29b-3p	7	3	**2,3**						
	hsa-miR-103a-3p	22	10	**2,2**						
	hsa-miR-103b	22	10	**2,2**						
	hsa-miR-7-5p	19	9	**2,1**						

### Abundance of rRNA-derived fragments is comparable in SIV virion and mock preparations

**Figure 3:**
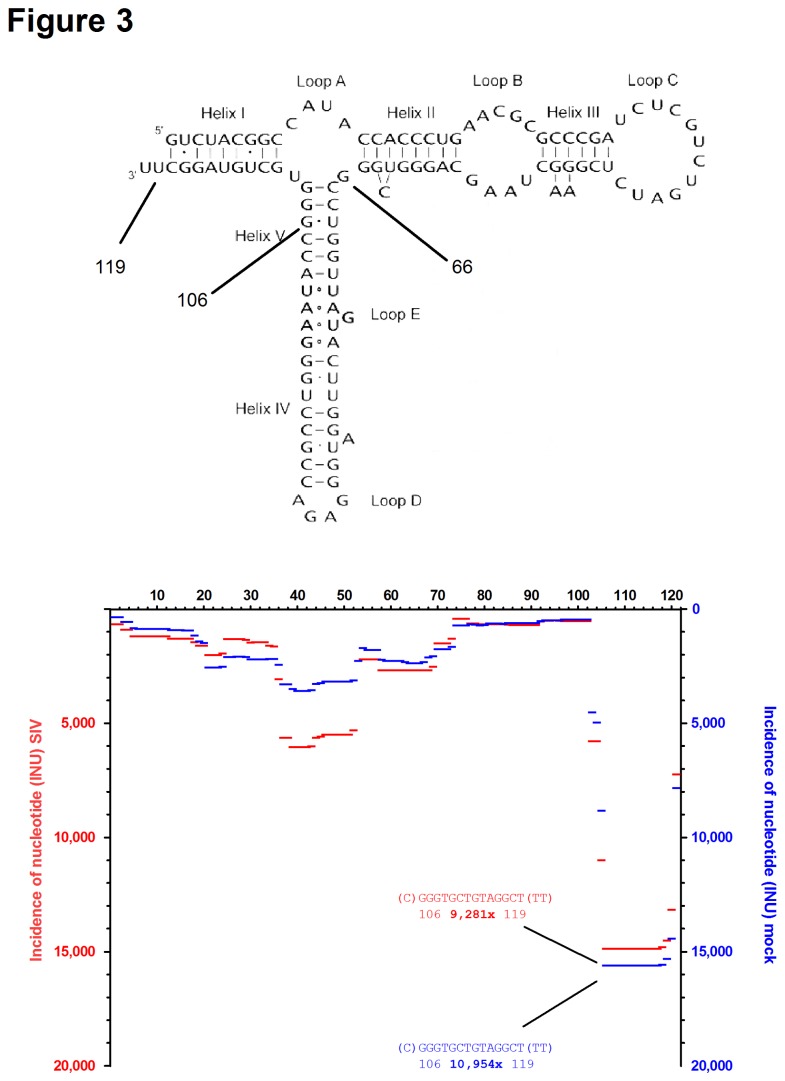
Fragment pattern of 5S rRNA. Sequence reads matching human 5S rRNA (Acc. no. V00589) were plotted according to their INU along the primary sequence. INUs from the SIV-virion sample (red, left y-axis) and the mock-treated sample (blue, right y-axis) are plotted on the same graph. The most abundant sequence reads (nt 106-119) are indicated with the number of identical reads given in bold. Note that sequences derived from the 5S rRNA hairpin (nt 66-108) are underrepresented.

Beside vsRNAs and miRNAs, we also identified other classes of non-coding RNAs that were present in both samples. As known from many other studies, fragments derived from ribosomal RNAs are highly abundant in small RNA preparations. Beside numerous fragments derived from 28S, 18S and 5.8S rRNA we also detected 24,559 and 21,827 small RNA fragments derived from 5S rRNA in SIV-containing and mock-infected cell culture supernatants, respectively. The fragment pattern was visualised by plotting the INUs against the nucleotide sequence of human 5S rRNA (Figure **3**). As with the vsRNAs from SIV, the fragments were not randomly distributed along the primary sequence. Interestingly, nucleotides that fold into a pre-miRNA-like structure, e.g. nt 66-106, were underrepresented, whereas the 3‘-end of 5S rRNA was sequenced ~10,000 times. Overall, our analysis demonstrated that both, the total abundance of 5S rRNA-derived fragments and the distribution of the fragments along the primary sequence were very similar in the two samples. Comparable fragment numbers and arrangement patterns were also seen for 5.8S, 18S and 28S rRNA (Figure **S3**). We therefore concluded that the small RNA content in the SIV and mock sample could be compared in a quantitative manner.

### High abundance of 7SL RNA-derived fragments in SIV virions

**Figure 4:**
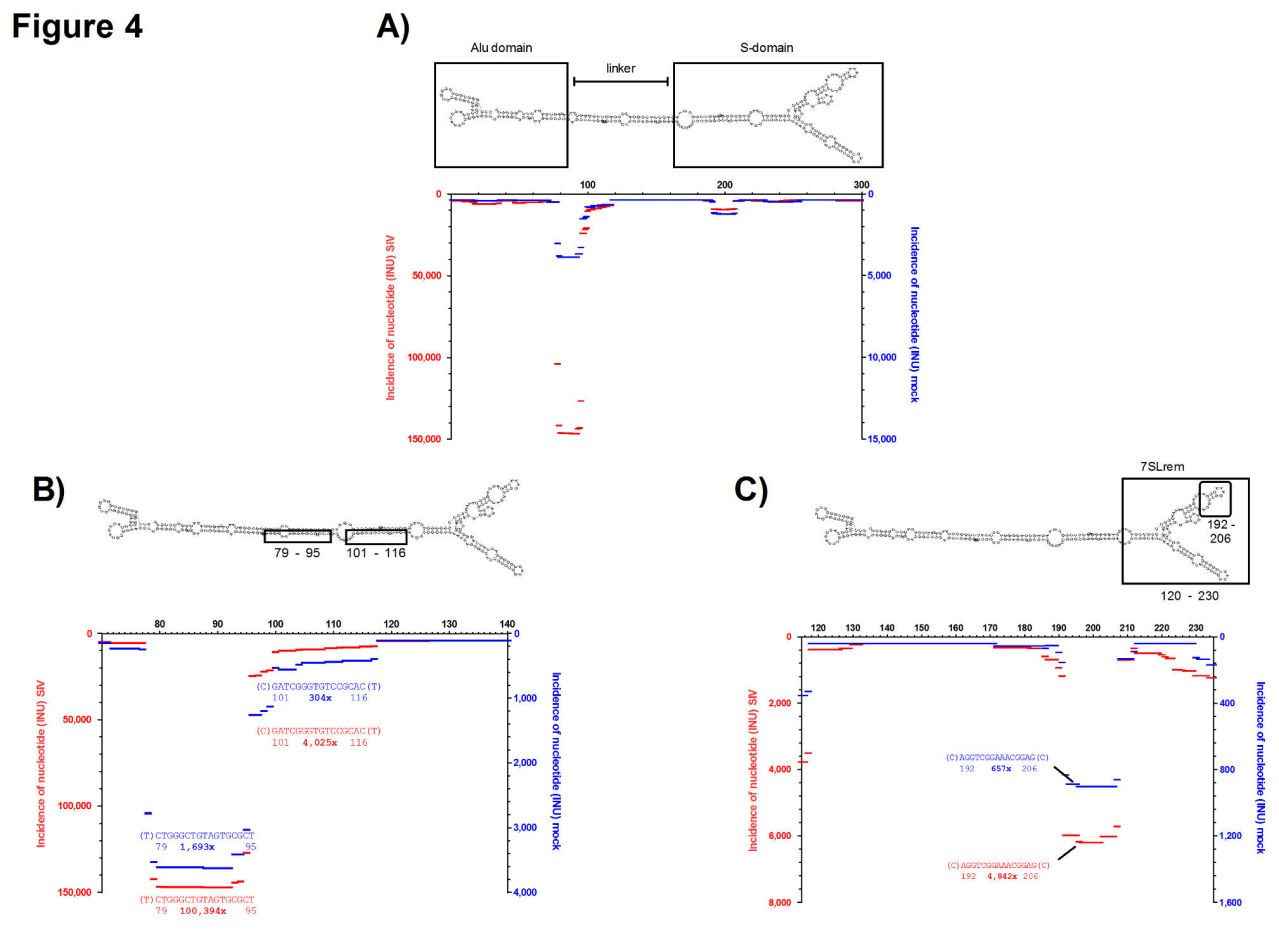
Fragment pattern of 7SL RNA. INU calculated from 160,274 (SIV) and 4,941 (mock) sequence reads were mapped on the 7SL RNA (Acc. no. NR_002715.1). INUs for fragments derived from SIV-virion containing (red, left y-axis) or mock-treated cell culture supernatants (blue, right y-axis) are plotted along the primary sequence. **A**) Total fragment pattern of 7SL fragments. Detailed maps of 7SL fragments are shown in **B**) (nt 70-140) and **C**) (nt 115-235). The most abundant fragments are shown together with their relative positions. The number of identical reads is given in bold. Secondary structure of 7SL according to RNAfold [33], domains, fragment topology and position of 7SLrem [17] are printed above the figures. Note that, for comparative reasons, the scale for the y-axes are different in **A**, **B** and **C**..

Another large proportion of sequence reads matched to the non-coding RNA 7SL. 7SL RNA is a component of the signal recognition particle (SRP) ribonucleoprotein that guides secretory proteins to the endoplasmic reticulum and is known to reside in retroviral particles [15]. A recent publication provided evidence that an endoribonucleolytic fragment, referred to as 7SL remnant (7SLrem), resides within HIV-1 virus-like particles [17]. Figure **4A** shows the distribution of RNA fragments that matched 7SL RNA. Overall, the incidence of 7SL RNA fragments was more than 30 fold higher in SIV compared to the mock sample (160,274 vs. 4,941 sequence reads). As seen for vsRNAs and rRNA, the incidence of individual 7SL-derived fragments differed largely along the primary sequence. In particular, a fragment that matched to the linker region between the Alu- and the S-domain of 7SL [36] was sequenced more than 100,000 times in the SIV-sample (Figure **4B**). In comparison, the corresponding sequence was only detected ~1,700 times in mock-infected cells, arguing for a specific enrichment of this fragment in SIV virions. Unexpectedly, this highly abundant fragment did not overlap with the described 7SLrem fragment that had been detected in HIV-1 virions. Nevertheless, we also found a short fragment from the 7SLrem region that was overrepresented in the SIV sample (Figure **4C**). In summary, the detected overrepresentation of 7SL RNA-derived sequences in the SIV sample was reassuring that our approach could be used to identify SIV virion-incorporated small RNAs.

### Unequal distribution of tRNA^Lys^-derived fragments

**Figure 5:**
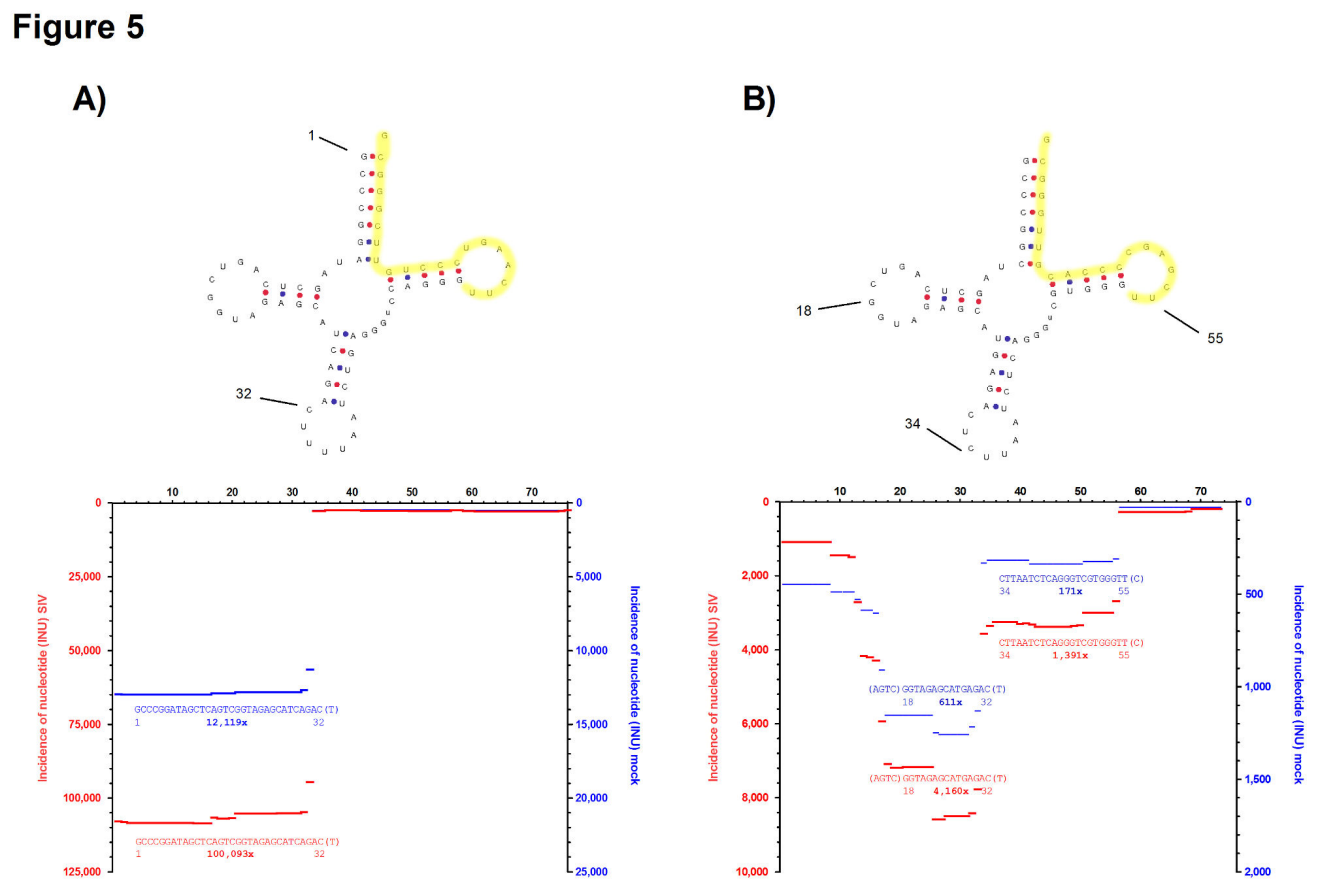
Fragment pattern of tRNA^Lys^ and tRNA^Lys^ isoacceptors. INUs were mapped on human tRNA^Lys3^ (**A**, Acc. no. M17621) and tRNA^Lys1,2^ (**B**, Acc. no. M33940). INUs for fragments derived from SIV-virion containing (red, left y-axis) or mock-treated cell culture supernatants (blue, right y-axis) are plotted along the primary sequence. For annotation, the mature tRNA sequences were used, i.e. nt 1 corresponds to nt 116 in Acc. no. M17621 and nt 961 in Acc. no. M33940, respectively. The most abundant fragments are shown together with their relative positions. The number of identical reads is given in bold. Secondary structure prediction, printed above the plot, was performed using tRNAscan-SE [67]. The sequence region mapping to the PBS region of SIV _mac_239 is labeled in yellow. Note that the scales of the y-axes differ in (**A**) and (**B**).

We also identified a variety of fragments that matched to tRNA^Lys3^. In the SIV sample, more than 100,000 sequence reads mapped to tRNA^Lys3^ whereas only ~12,000 could be found in supernatants from mock-infected cells (Figure **5A**). This is in line with the current view of tRNA^Lys3^ incorporation into virions. However, almost 95% of these reads were represented by a single, 32-33 nt long tRNA-derived fragment (tRF) (also see Figure **S4**) that mapped to the 5‘-portion of tRNA^Lys3^ adjacent to the anticodon sequence. In addition, we expected to find a high proportion of tRNA^Lys3^-derived fragments that were fully complementary to the PBS of SIV. However, we only detected 363 and 14 tRFs, in infected and uninfected supernatants, respectively (Figure **5A**).

We also identified sequence reads that matched tRNA^Lys1,2^ isoacceptors (Figure **5B**). Although tRNA^Lys3^ and tRNA^Lys1,2^ share a high degree of sequence identity, none of the sequence reads matched to both tRNA isoacceptors. Our analysis revealed that the total number of tRNA^Lys1,2^-derived fragments were sevenfold more abundant in SIV compared to mock-controls (10,367 *vs.* 1,443). Concordantly with tRNA^Lys3^, most tRNA-fragments matched to the 5’-portion of tRNA^Lys1,2^, while the incidence in the PBS region was comparably low. Since binding of tRNA^Lys^ to the PBS must be expected, it remains elusive why the corresponding fragments were underrepresented. Interestingly, the size distribution of the tRNA^Lys1,2^ derived fragments was markedly different. Here, approximately 30% were in the range of 16-17 nt, 40% were between 20-23 nt and only 20% were 30-33 nt in length (Figure **S4**). In summary, our analysis showed that tRNA^Lys^-derived fragments are incorporated into SIV virions, however, the detected fragments of the isoacceptors showed a markedly different fragment pattern and only very rarely matched the SIV primer-binding site (PBS) region.

### Evidence for SIV virion association of non-coding RNA snaR-A14

**Figure 6:**
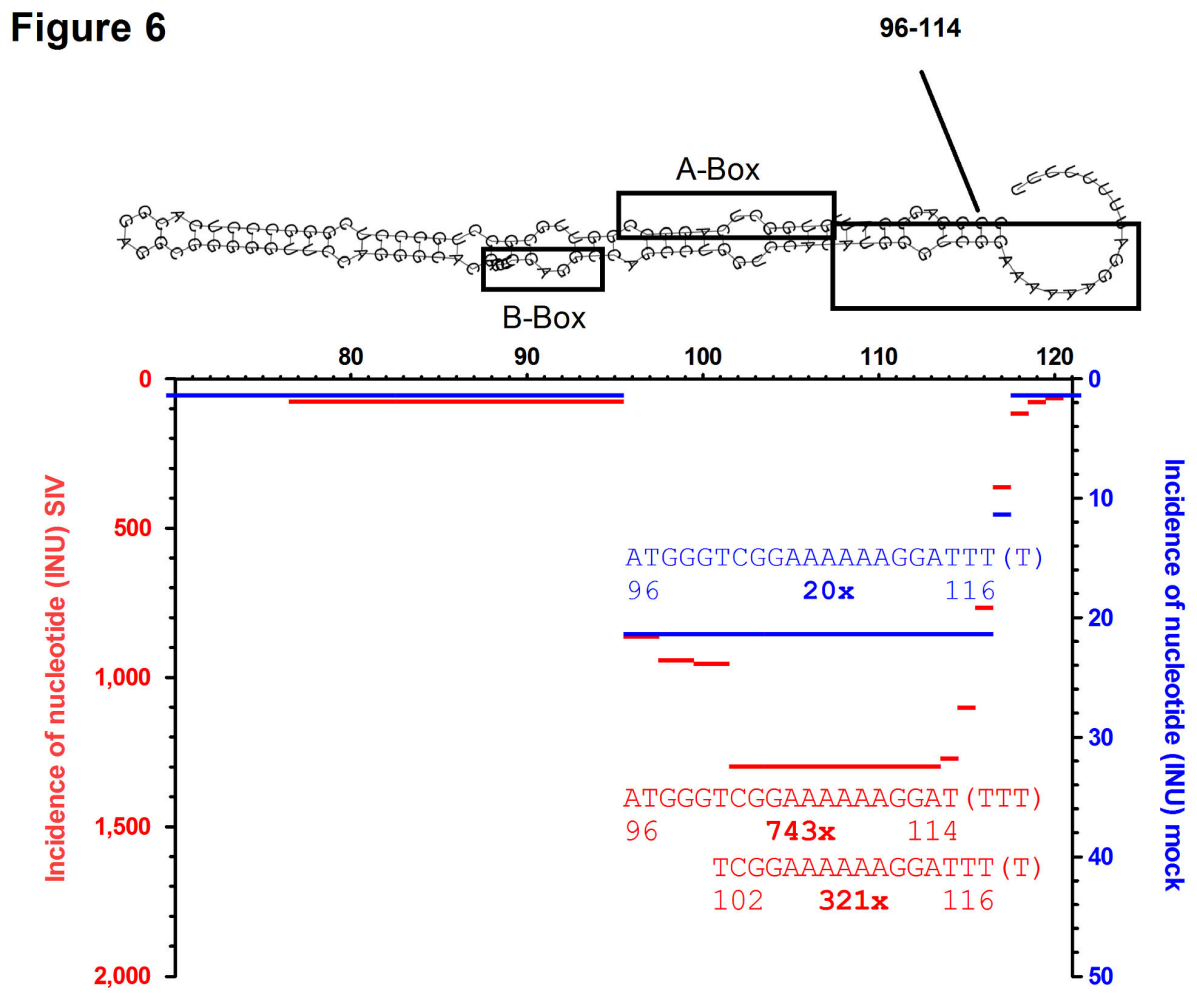
Fragment pattern of snaR. INUs were mapped on human small ILF3/NF90-associated RNA A14 (SNAR-A14, Acc. no. NR_24242.1). INUs for fragments derived from SIV-virion containing (red, left y-axis) or mock-treated cell culture supernatants (blue, right y-axis) are plotted along the primary sequence. The most abundant fragments are shown together with their relative position and the number of identical reads in bold. Secondary structure of snaR-A14, that was predicted with RNAfold [33], is given above. The annotation of A-Box and B-Box is according to [38]. Note that this sequence is identical in SNAR-A3, A4, A5, A6, A7, A8, A9, A10 and A11.

Further BLAST analysis revealed one additional non-coding RNA, snaR-A14, which was more abundant in the SIV sample. Here we identified more than 1,000 fragments that matched to the family of small ILF3/NF90-associated RNAs (snaR) (Figure **6**). The RNA polymerase III-transcribed members of this family bind to the nuclear factor 90 (ILF3/NF90) protein [37], which is involved in multiple cellular functions. SnaR RNAs are highly structured non-coding RNAs of ~120 nt in length that reside in the cytoplasm and are associated with ribosomes [38]. Using the INU approach we found that a 3’-fragment of snaR-A14 was 50 fold more abundant in SIV compared to mock (1064 *vs.* 20 reads), arguing that snaR-A14 is also incorporated in SIV virions.

### Small nucleolar RNAs U2 and U12 are less abundant in SIV virion preparations as compared to controls

**Figure 7:**
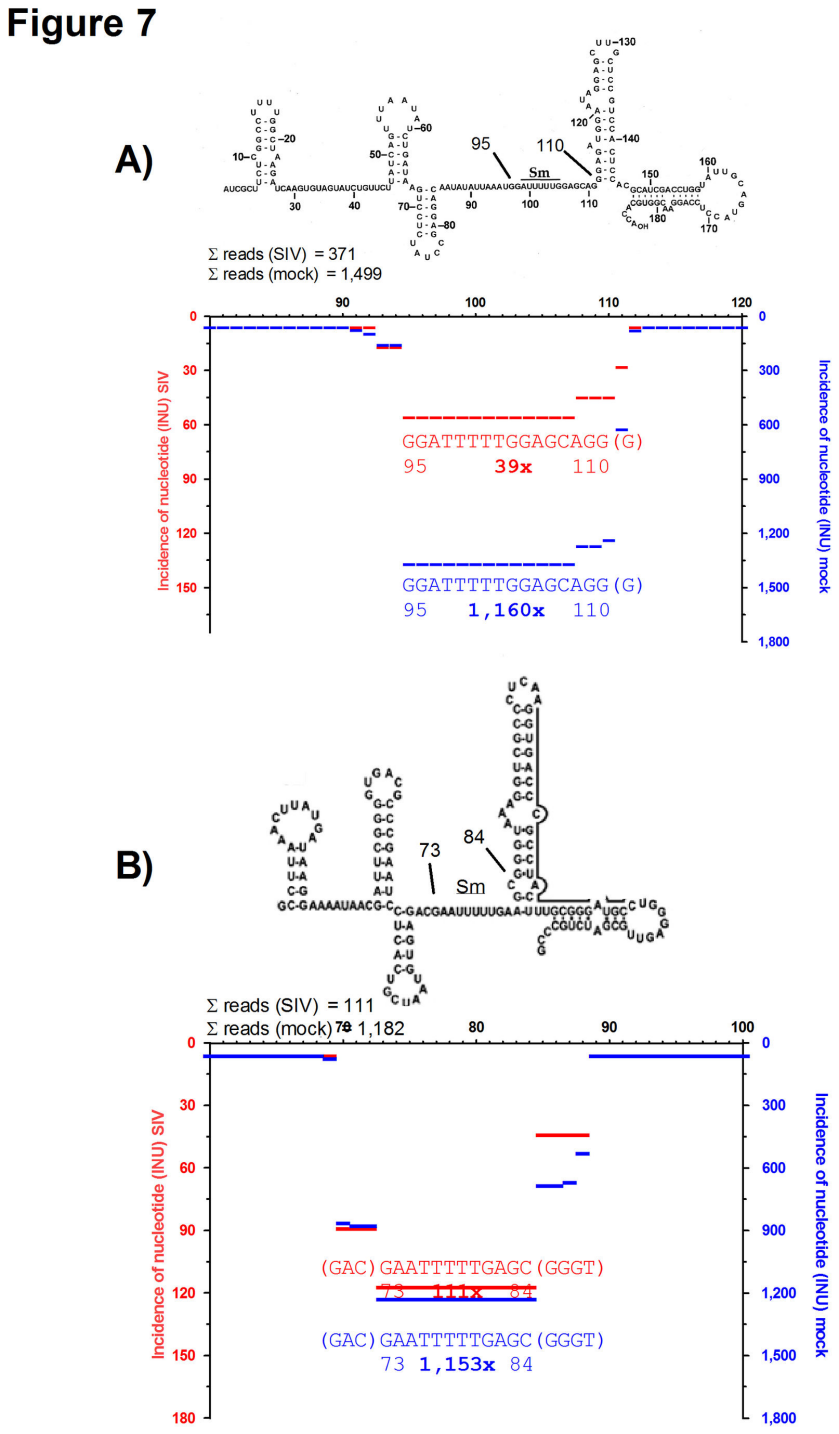
Fragment pattern of U2 and U12 snRNA. INUs were mapped on human U2 (**A**, Acc. no. X59360) and U12 (**B**, Acc. no. J04119). INUs for fragments derived from SIV-virion containing (red, left y-axis) or mock-treated cell culture supernatants (blue, right y-axis) are plotted along the primary sequence. The most abundant fragments are shown together with their relative position and the number of identical reads in bold. Secondary structure prediction for U2 and U12 was copied from [68] and [69], respectively. The Sm-regions are indicated. Note that the incidence of fragments in the mock-preparation is more than tenfold higher compared to SIV.

We also found fragments that matched to small nuclear RNAs (snRNAs) that function in the spliceosome. The most abundant fragments were derived from U1 (RNU1), U2 (RNU2), U12 (RNU12) and U33 (RNU33, SNORD33) known to be involved in the cleavage of divergent classes of low-abundance pre-mRNA introns. In total, the number of reads that matched snRNA U2 were higher in the mock compared to the SIV preparation (1,499 *vs.* 371). In particular, sequences that matched nt 95-111 were 30 fold more abundant in the mock sample (Figure **7A**). A comparable enrichment was also seen for snRNA U12-derived sequences that were 10 fold more abundant in the mock preparation compared to the SIV sample (1182 vs. 111 reads) (Figure **7B**). The most abundant fragments in both, snRNA U2 and U12, coincidentally covered the Sm site that is flanked by stem-loop structures. The Sm site element is a short, single-stranded nucleotide sequence with a consensus region [39,40] that binds to so-called Sm ribonucleoproteins, key factors that coordinate cleavage and rejoining of pri-mRNAs during splicing. In comparison, the incidence of snRNA U1- and U33-derived fragments were similar in the SIV and mock sample (1573 vs. 1101 reads and 1698 vs. 774, respectively, Figure **S5**). Thus, snRNA species depletion in the SIV sample was not a general phenomenon of small nucleolar RNAs, but restricted to snRNAs U2 and U12. 

## Discussion

Our study aimed at generating an atlas of non-coding RNAs incorporated into retroviral virions using SIV _mac_239 as a model virus. For our approach we have analyzed the small RNA content of SIV virions that was compared to a preparation derived from mock-infected cells. It has to be mentioned that the interpretation of the results is limited by the technical preparation of the samples. Treatment of PEG-precipitated particles with high amounts of RNases, which has also been used by others [15,26,41], aimed to degrade small RNAs that were not protected by virions or vesicles. Concomitantly, it has to be assumed that RNases may have entered leaky particles and led to partial degradation of their content. However, using two different methods we could not find significant differences between nuclease-treated and untreated samples, indicating that only a minor part of small RNAs may have resulted from the experimental procedure.

Another limitation of the experimental setup results from the chosen protocol, which was applied to enrich virions and vesicles. Using a crude PEG precipitation step we were able to retrieve high amounts of virions, but with only moderate purity. Thus, a considerable amount of small RNAs in the virion preparation may not be derived from viral particles, but may originate from gRNA-free particles or exosomes. Other preparation protocols, e.g. velocity gradient-based methods, have been successfully used to separate virions from vesicles [42], however, the comparably low yield of virions and small RNAs thereof limits the generation of libraries for ultra-deep sequencing that may severely decrease the number of reads impairing quantitative comparison. Thus, the used approach conveys certain limitations, but the use of alternative methods would only shift uncertainties from one end to another. Direct comparison of the mock to the SIV sample enabled us to estimate the reliability of our results and within the mentioned limitations, several findings support the validity of our interpretations:

(I) The ratio and spatial distribution of 5S, 5.8S, 18S and 28S rRNA-derived fragments were in the same range in the SIV and the mock sample, indicating that the preparations had comparable quality and quantity.
(II) The described vsRNAs derived from SIV were quantitatively different from the more or less randomly distributed fragments that were found at a much lower frequency along the genomic sequence, which is in agreement with comparable data from other viruses [3].
(III) Sequence reads that matched tRNA^Lys3^ and tRNA^Lys1,2^ were ~8 fold higher in the SIV sample, which is in line with previous observations in HIV-1 particles [43,44].
(IV) The number of reads that matched 7SL RNA-derived fragments was 30 times higher in the SIV virion preparations, confirming the known constituent of retroviral particles [17,45].

The fact that the enrichment of distinct groups of ncRNAs is in concordance with published data for retroviral particles indicates that our approach can be used to characterize the small virionome of SIV virions reliably.

### Identification of vsRNAs in SIV virion preparations

Our analysis showed that a small set of highly abundant vsRNAs can be isolated from SIV virion preparations. Evidence accumulates that RNA viruses can give rise to a variety of vsRNAs [3], however, only very few have been found to be functional so far [6,46]. The existence of functional, lentiviral-derived vsRNAs has been discussed controversially. While some investigators failed to detect any HIV-1 derived small RNAs [47,48], others have claimed their identification [4,9,49]. A very recent study reported that more than 1% of the total small RNA content derived from HIV-1 particle preparations matched the gRNA of the lentivirus [8]. In addition, a set of HIV-1 derived vsRNAs were also identified in HIV-1 infected cells [45]. We now provide evidence for the existence of vsRNAs in the closely homologous lentivirus SIV. Our analysis showed that some vsRNAs were sequenced more than 12,000 times, thereby representing more than 1% of the total number of sequence reads in the SIV preparation. This is in line with the occurrence of vsRNAs in other RNA viruses, where small RNA fragments appeared as „hot spots“ [3,45]. However, using secondary structure predictions we did not find evidence that the SIV-derived vsRNAs were derived from pre-miRNA-like precursors. Thus, these vsRNAs may not have been generated by the cellular RNAi machinery. This is in accordance with previous observations that the absence of Dicer in RNA-virus infected cells markedly reduces the abundance of miRNAs, but only marginally affects the number of detectable vsRNAs from RNA viruses [3]. It remains to be explored whether these vsRNAs exert biological functions in the virion or play an immunomodulatory role in the host cell after viral entry.

### Incorporation of host small RNA fragments in SIV virions

Consistent with previous studies we have identified a number of host-encoded small RNAs in supernatants from SIV- and mock-infected cells. The total amount of cellular miRNAs that was detected in our preparations was markedly low, and only very few showed considerable enrichment in the SIV-sample. The most prominent candidate, hsa-miRNA-10b, has been described to promote metastasis formation [50] and glioma growth [51], but has not been reported in the context of retroviral biology. Using the miRanda prediction tool [52], we found two putative binding sites for miR-10b on the SIV genome, but only when using relatively weak filtering options. It remains to be seen by experimental validation, if those sites are real effective targets of miR-10b, since computational target prediction tools alone cannot provide sufficient evidence. Comparison of the identified miRNAs to those that were detected in HIV-1 virion preparations [45] showed no overlap with our results. Whether or not this is due to the experimental setup or the used virus remains open. On the opposite, we found a considerable number of fragments that matched known retrovirion-incorporated small RNAs, such as 7SL RNA and tRNA^Lys^ isoacceptors [13,15,17,43]. These were markedly enriched in the virion-containing sample by a factor 8-10. Selective incorporation of non-lysosyl tRNAs into HIV-1 is known for long [13], however, the exact function of these tRNAs remains elusive. We now describe the identification of tRNA-derived fragments into virions of the closely related SIV, which complements our current picture of virion composition, but does not shed light on its role. However, increasing evidence indicates that tRFs are functional RNAs, rather than degradation products derived from highly abundant RNA species. Recent studies showed that asymmetric processing of tRNAs is a common mechanism used by eukaryotes [53,54] and that some tRNA-derived small RNAs show a specific expression pattern that exert biological functions, such as the induction of cell proliferation or correlate with miRNA and siRNA silencing activities [55-59]. In particular, a 3’-terminal fragment derived from tRNA^Lys3^ (PBSncRNA) has been found to associate with Argonaute-2 (Ago2) protein through interaction with the PBS sequence in HIV-1-infected cells [9], where it was shown to modulate viral replication. While PBSncRNA functions within infected cells, we now found that the left-over sequence from tRNA^Lys3^ is incorporated in the virion. Whether or not release of this 1-33 tRNA^Lys3^ moiety into newly infected cells exerts any function remains unknown.

In addition to tRNA^Lys^-derived fragments and tRFs derived from other cellular tRNAs (see Table **S2** and Figures **S6** and **S7**), we also identified one previously unknown fragment that was derived from the non-coding RNA snaR-A14. This small, Pol III-transcribed RNA is associated with nuclear factor 90 (NF90) and is highly transcribed in immortal cell lines as well as in testis [37]. It has been proposed that snaR has evolved from primate-specific short interspersed elements (SINE), but to date no functional data are available. Interestingly, NF90 has been recently described as a regulator of HIV-1 replication during both productive infection and induction from latency [60], which might be connected to our finding.

### Depletion of U2 and U12 snRNA fragments in supernatants from SIV-infected cells

While our approach aimed at identifying virion-enriched small RNAs, we also detected small RNAs that were more abundant in supernatants from mock-infected cells, which appears puzzling at first sight. As described above, the RNA content of supernatants from mock-infected cells should be enriched in exosomes that are released into the cell culture medium by the used human T-cell line C8166. Accordingly, sequence reads derived from the mock-sample mirror the content of small RNAs that reside in these exosomes. In comparison, supernatants from SIV-infected cells should harbour virions, but also contain exosomes. Thus, the lower abundance of a small RNA in the SIV-virion containing sample can be explained either by a lower amount of co-isolated exosomes or by alterations of the exosomal content. Accumulating evidence indicates that exosomes play pivotal roles in physiological and pathological settings, including antigen presentation and the transport of infectious agents [61]. Cell recognition molecules on the exosomal surface enable selective targeting of recipient cells. Furthermore, exosome-embedded mRNAs and miRNAs can be shuttled into recipient cells and modulate their function [62-64]. Regarding the immune system, it has been found that T-cells can unidirectionally transfer exosomal information to antigen presenting cells [65]. Furthermore, an exosome-mediated transfer of Epstein-Barr virus (EBV)-encoded miRNAs leads to repression of EBV target genes in recipient B-cells [66]. Thus it appears that exosomes can convey RNA-encoded information systemically, which has gained broad interest over the past couple of years.

Here, we provide the first evidence that fragments derived from snRNAs U2 and U12 were 30 fold and 10 fold less abundant, respectively, in an SIV virion preparation. According to the arguments given above, this finding might reflect a reduction in the release of exosomes by SIV-infected cells or indicate that the small RNA content of these exosomes has changed. In either case, our data support the hypothesis that retroviral infection can affected the machinery of cell-to-cell communication. It is therefore tempting to speculate that retroviruses can influence the immune system by altering the intercellular communication. This would be very difficult to verify experimentally, since the target cells probably might not be infected by the retrovirus.

In summary, our study provides an atlas of vsRNAs and cellular small RNA-derived fragments that are selectively packaged into SIV virions. The particles not only contain tRNA^Lys^, and 7SL RNA, but also NF90-binding RNA and snaR-A14, that are described here for the first time. Moreover, our data suggest that SIV-infection can lead to changes in the small RNA content of exosomes that might affect the intercellular communication of the immune system. Thus, the analysis of the putative function of these small RNAs may reveal new insights into retroviral-induced pathogenesis.

## Supporting Information

Figure S1 **Treatment of DNase/RNase does not affect virion integrity.** Virion preparations (PEG pellets) were treated with or w/o DNase/RNase as outlined in Material and Methods. Quantification of isolated viral RNA by QPCR +/-SD (open bars) refer to the left y-axis, the corresponding infectious titer (belted bars) refer to the right y-axis. Copy numbers were not significantly different (n.s.) between the three samples (OneWay ANOVA, Bonferroni’s Multiple Comparison Test, p > 0.05).
(TIF)

Figure S2 **Low abundance SIV-derived vsRNAs.** High resolution map of Figure 1 with sequence reads that mapped SIV _mac_239 with 500 INUs or less.
(TIF)

Figure S3 **Distribution of rRNA-derived fragments.** INUs were plotted along their position on the human sequence of 28S (**A**, Acc. no. M11167.1), 5.8S (**B**, Acc. no. NR_003285) and 18S rRNA (**C**, Acc. no. NR_046235.1; nt 3512-5734). INUs for fragments derived from SIV-virion containing (red, left y-axis) or mock-infected cell culture supernatants (blue, right y-axis) are plotted along the primary sequence.
(TIF)

Figure S4 **Fragment size distribution.** Total number of fragments of SIV (grey), 28S rRNA (yellow), 7SL RNA (green), tRNA^Lys3^ (red) and tRNALys^1,2^ (blue), respectively, were set to 100%. The relative abundance of fragments (in percent) are plotted against their fragment sizes.
(TIF)

Figure S5 **Fragment pattern of snRNAs** **U1 and U33**. INUs were mapped on human U1 (**A**, Acc. no. NR_004430.2), and U33 (**B**, Acc. no. X94599.1, nt 97-175). INUs for fragments derived from SIV-virion containing (red, left y-axis) or mock-treated cell culture supernatants (blue, right y-axis) are plotted along the primary sequence.
(TIF)

Figure S6 **Fragment pattern of tRNA-derived fragments (tRFs).** INUs of tRFs derived from tRNA^Gly^ (**A**), tRNA^Glu^ (**B**), tRNA^Asp^ (**C**), and tRNA^Ile^ (**D**) were plotted along the corresponding tRNA sequence. The most abundant fragments are shown together with their relative positions. The number of identical reads is given in bold. Secondary structure predictions that are printed above the plots were performed using tRNAscan-SE [67](67), the tRNA accession numbers are given in brackets.
(TIF)

Figure S7 **Fragment pattern of virion-enriched tRFs.** INUs of tRFs derived from tRNA^Thr^ (**A**), tRNA^Arg^ (**B**), tRNA^Met^ (**C**), tRNA^Asn^ (**D**), tRNA^Tyr^ (**E**), tRNA^Gln^ (**F**) and tRNA^His^ (**G**) from SIV and mock were plotted along the corresponding tRNA sequence. The most abundant fragments are shown together with their relative positions. The number of identical reads is given in bold. Secondary structure predictions that are printed above the plots were performed using tRNAscan-SE [67], the tRNA accession numbers are given in brackets. Note that tRNA^Thr^-derived tRFs (A) were exclusively found in SIV virions and that the scales of the y-axes differ in (B)-(F).
(TIF)

Table S1 **Complete list of miRNAs identified in virion- and mock preparations.**
(PDF)

Table S2 **Abundance of exosomal or virion-associated tRFs.**
(PDF)
