# Supplementary figures and images for: Mapping the Small RNA Content of Simian Immunodeficiency Virions (SIV)
Source: PLoS One. 2013 Sep 23;8(9):e75063. doi: 10.1371/journal.pone.0075063 (PMC3781035; doi:10.1371/journal.pone.0075063)

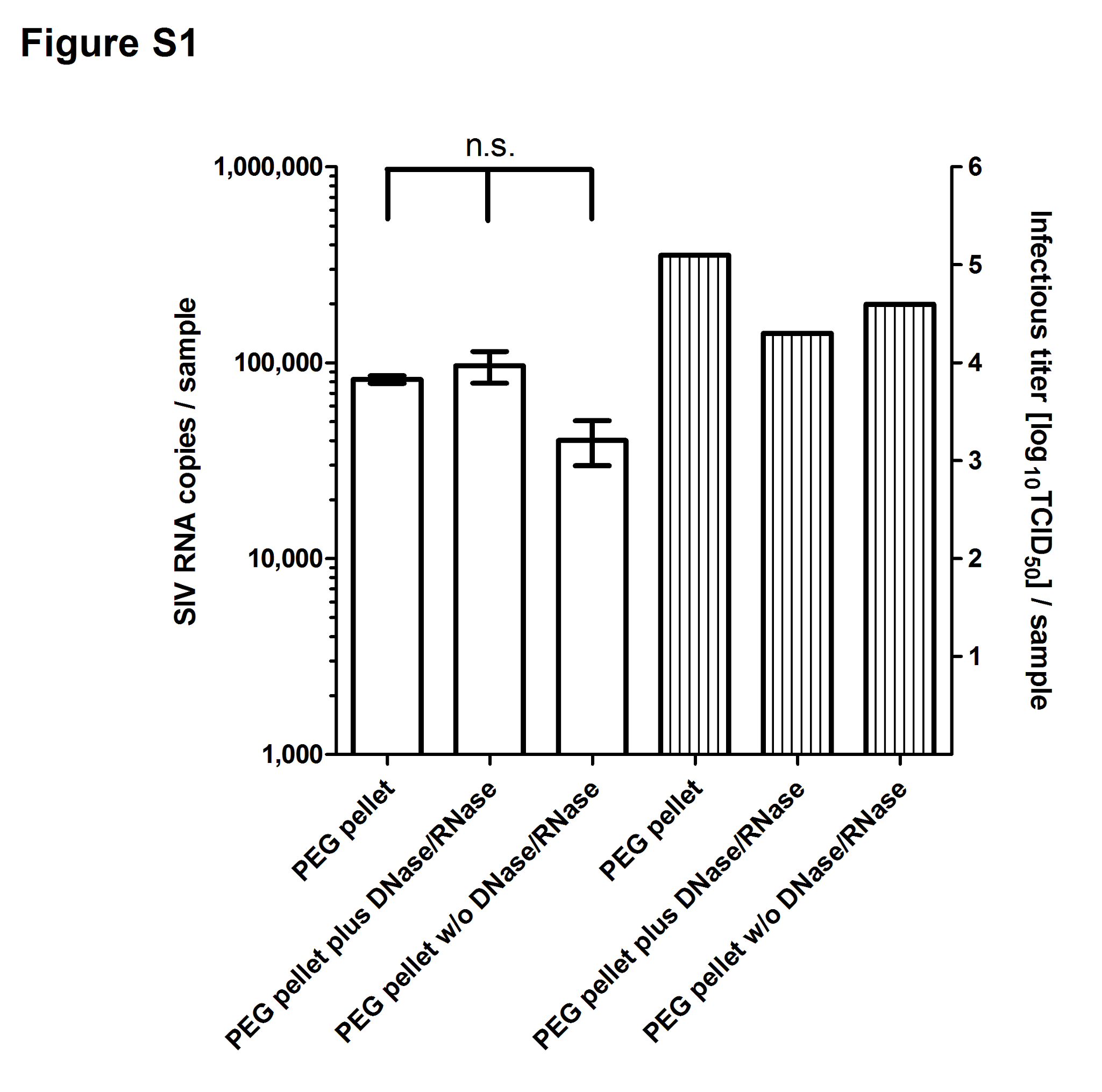

Supplement: Figure S1 — Treatment of DNase/RNase does not affect virion integrity. Virion preparations (PEG pellets) were treated with or w/o DNase/RNase as outlined in Material and Methods. Quantification of isolated viral RNA by QPCR +/-SD (open bars) refer to the left y-axis, the corresponding infectious titer (belted bars) refer to the right y-axis. Copy numbers were not significantly different (n.s.) between the three samples (OneWay ANOVA, Bonferroni’s Multiple Comparison Test, p > 0.05). (TIF) [file pone.0075063.s001.tif]

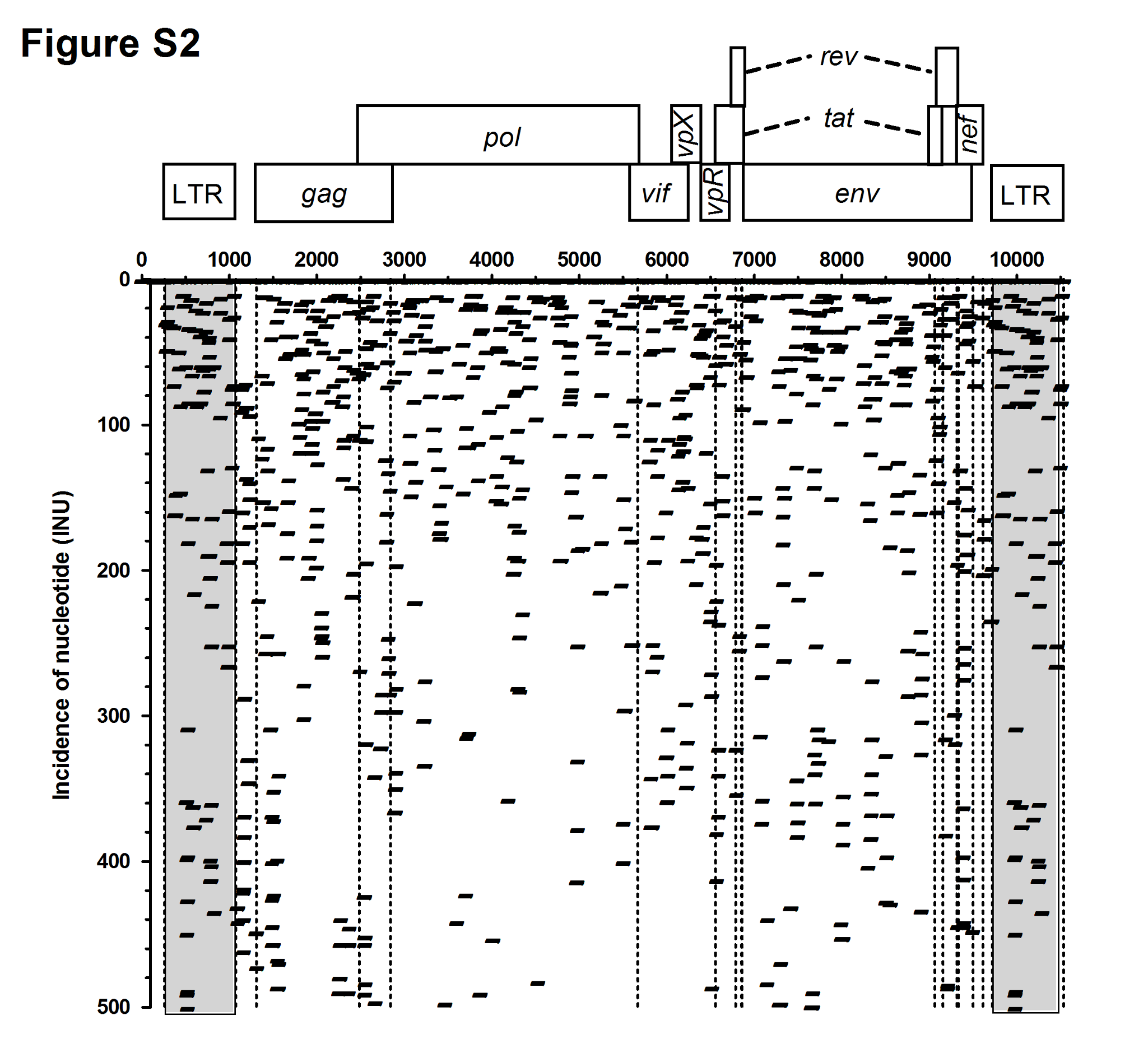

Supplement: Figure S2 — Low abundance SIV-derived vsRNAs. High resolution map of Figure 1 with sequence reads that mapped SIV mac239 with 500 INUs or less. (TIF) [file pone.0075063.s002.tif]

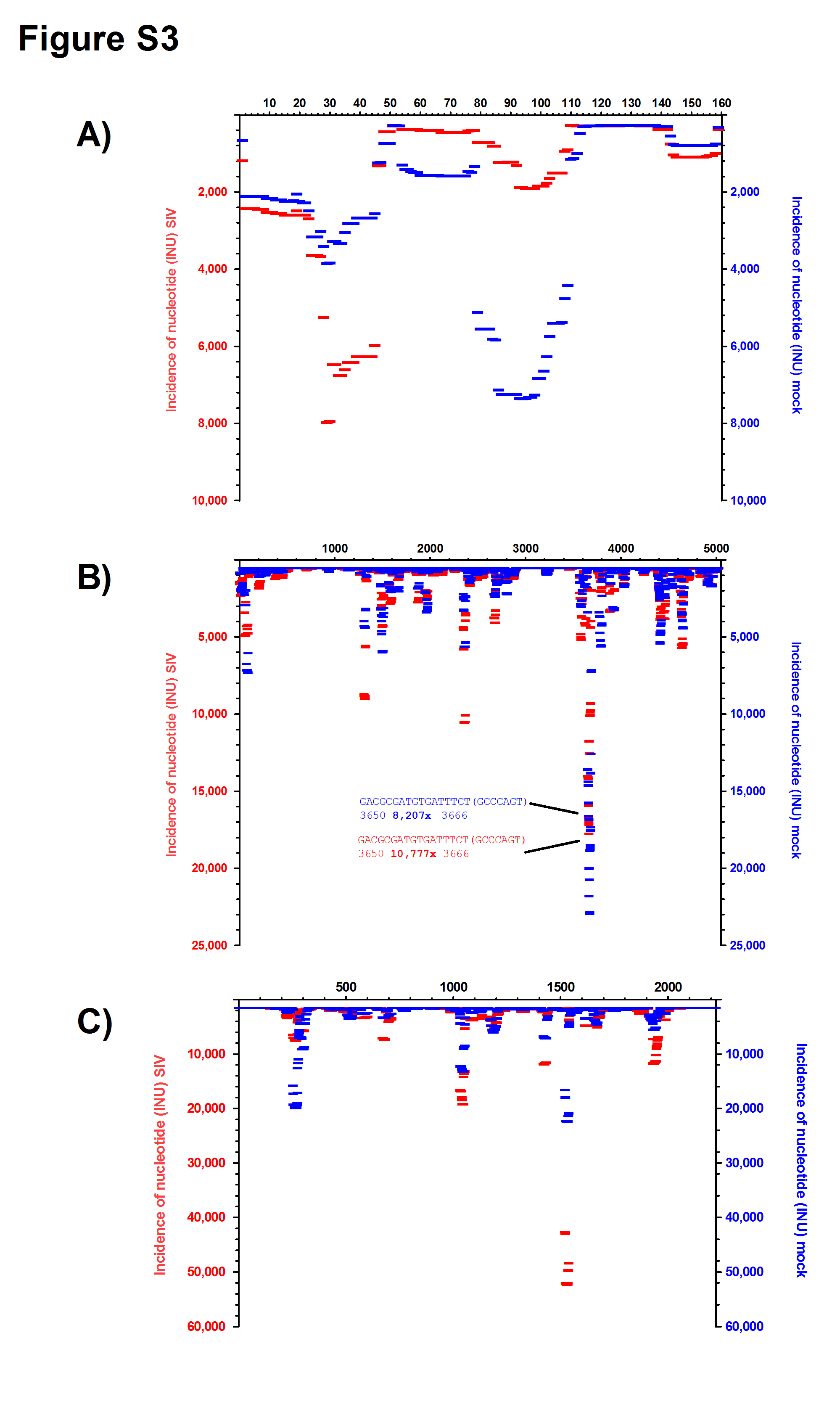

Supplement: Figure S3 — Distribution of rRNA-derived fragments. INUs were plotted along their position on the human sequence of 28S (A, Acc. no. M11167.1), 5.8S (B, Acc. no. NR_003285) and 18S rRNA (C, Acc. no. NR_046235.1; nt 3512-5734). INUs for fragments derived from SIV-virion containing (red, left y-axis) or mock-infected cell culture supernatants (blue, right y-axis) are plotted along the primary sequence. (TIF) [file pone.0075063.s003.tif]

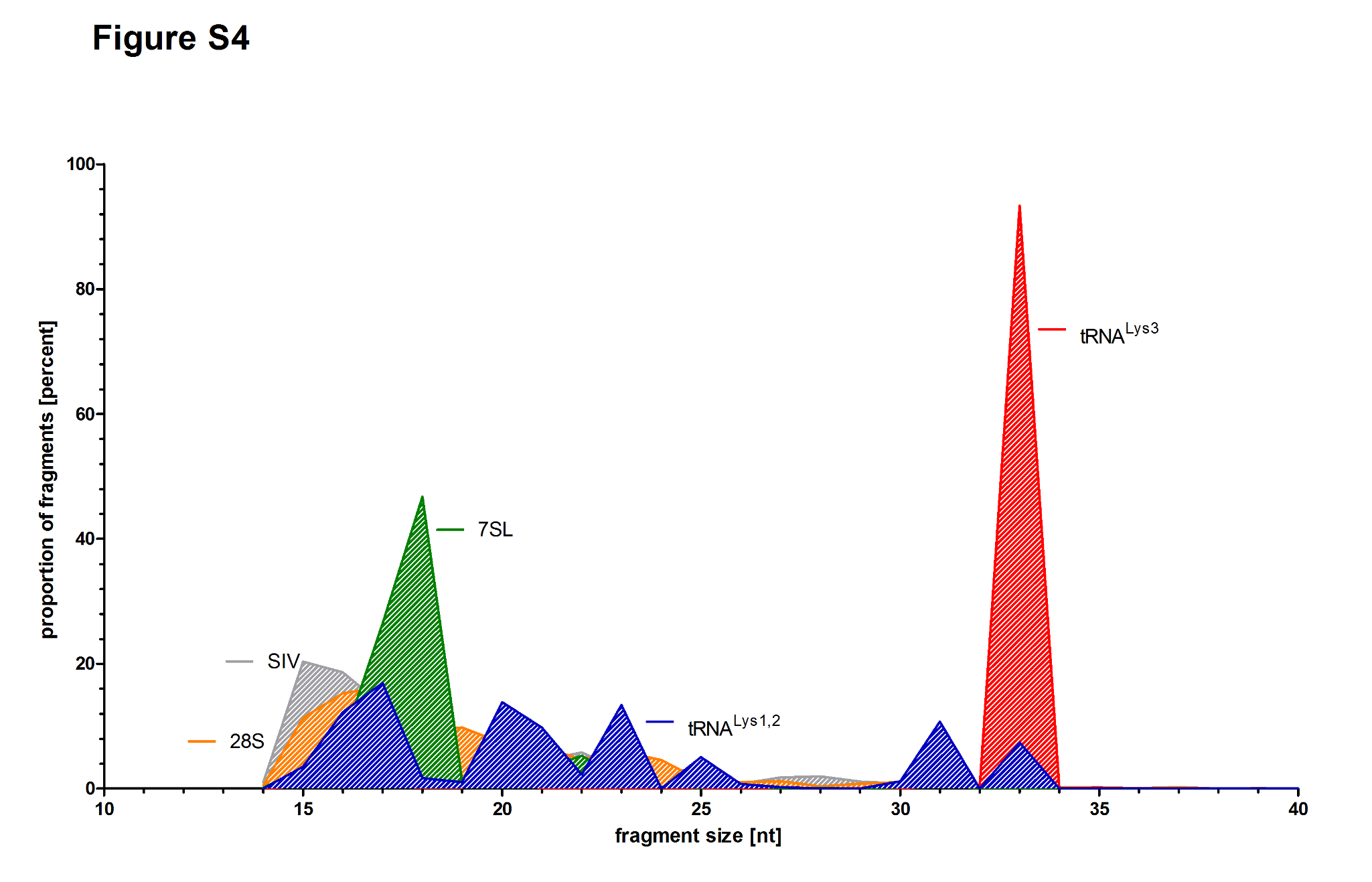

Supplement: Figure S4 — Fragment size distribution. Total number of fragments of SIV (grey), 28S rRNA (yellow), 7SL RNA (green), tRNALys3 (red) and tRNALys1,2 (blue), respectively, were set to 100%. The relative abundance of fragments (in percent) are plotted against their fragment sizes. (TIF) [file pone.0075063.s004.tif]

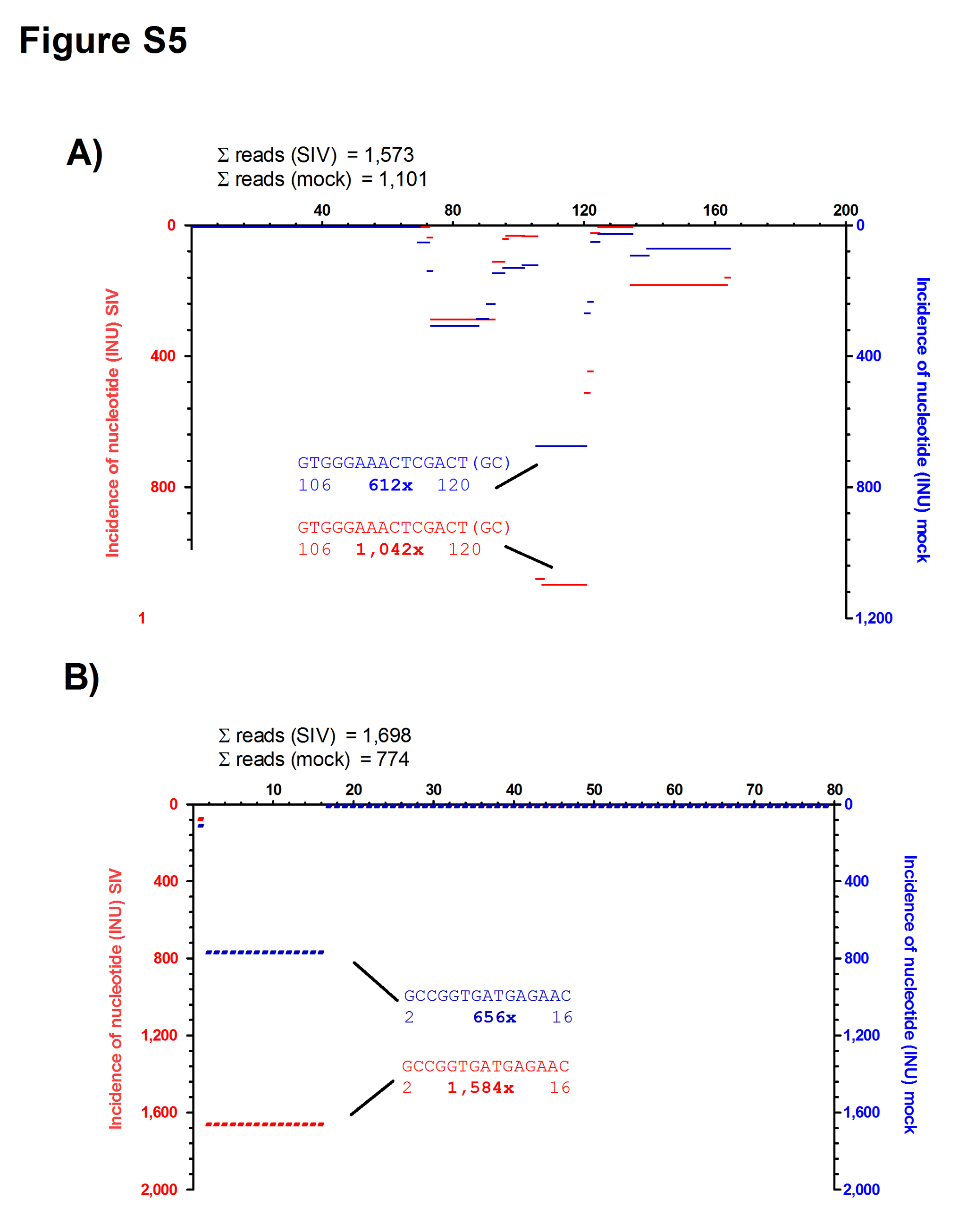

Supplement: Figure S5 — Fragment pattern of snRNAs U1 and U33. INUs were mapped on human U1 (A, Acc. no. NR_004430.2), and U33 (B, Acc. no. X94599.1, nt 97-175). INUs for fragments derived from SIV-virion containing (red, left y-axis) or mock-treated cell culture supernatants (blue, right y-axis) are plotted along the primary sequence. (TIF) [file pone.0075063.s005.tif]

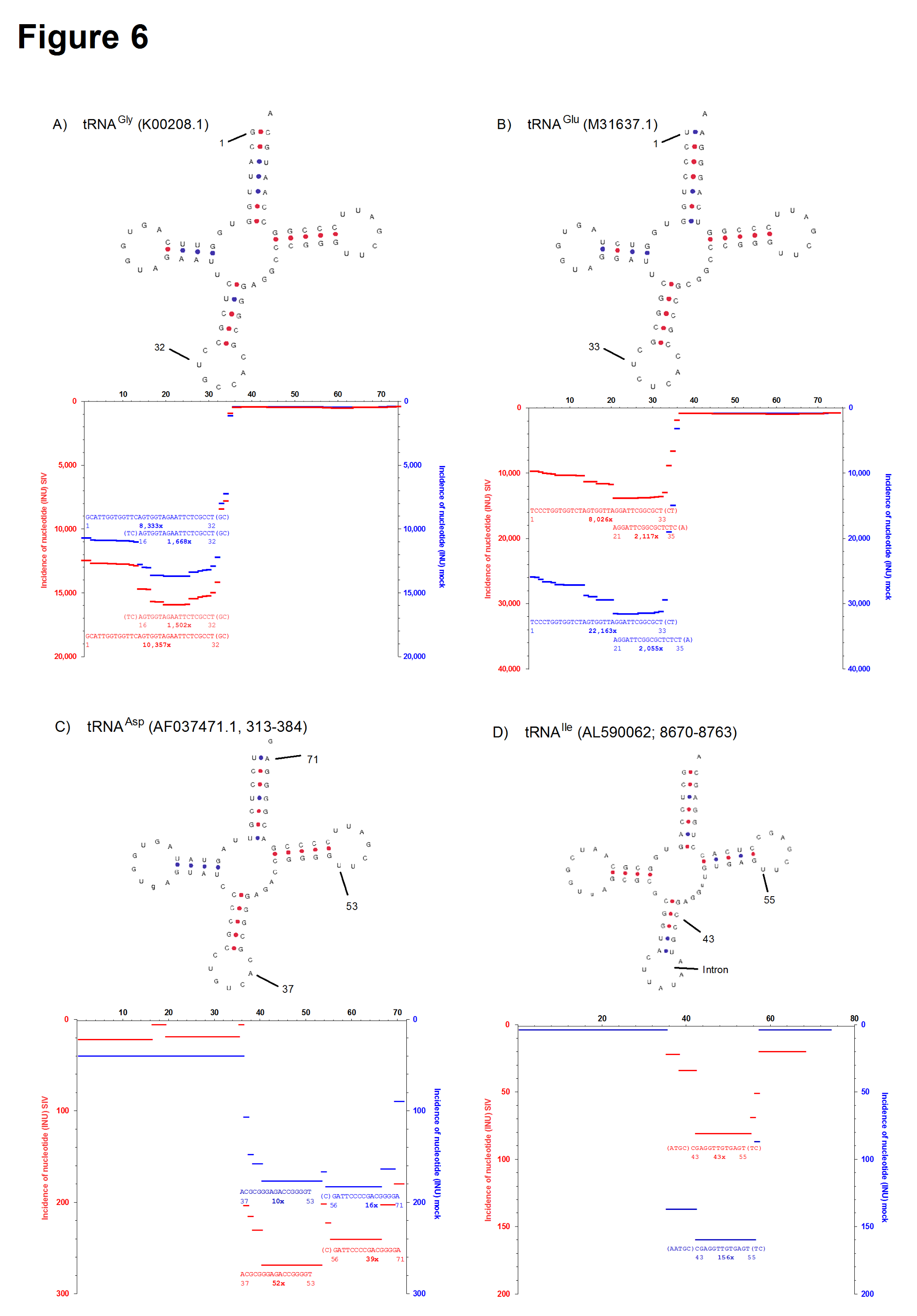

Supplement: Figure S6 — Fragment pattern of tRNA-derived fragments (tRFs). INUs of tRFs derived from tRNAGly (A), tRNAGlu (B), tRNAAsp (C), and tRNAIle (D) were plotted along the corresponding tRNA sequence. The most abundant fragments are shown together with their relative positions. The number of identical reads is given in bold. Secondary structure predictions that are printed above the plots were performed using tRNAscan-SE [67](67), the tRNA accession numbers are given in brackets. (TIF) [file pone.0075063.s006.tif]

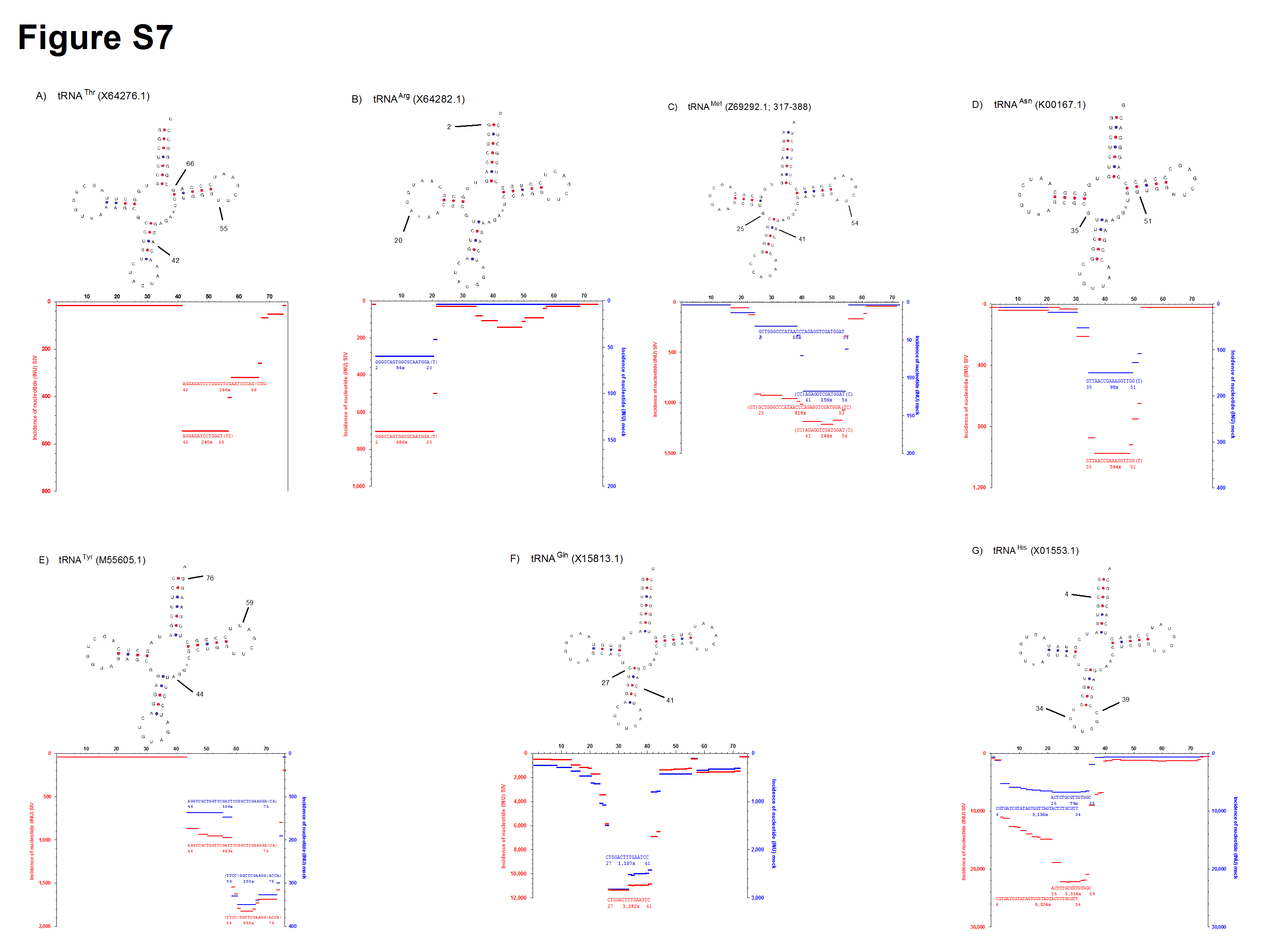

Supplement: Figure S7 — Fragment pattern of virion-enriched tRFs. INUs of tRFs derived from tRNAThr (A), tRNAArg (B), tRNAMet (C), tRNAAsn (D), tRNATyr (E), tRNAGln (F) and tRNAHis (G) from SIV and mock were plotted along the corresponding tRNA sequence. The most abundant fragments are shown together with their relative positions. The number of identical reads is given in bold. Secondary structure predictions that are printed above the plots were performed using tRNAscan-SE [67], the tRNA accession numbers are given in brackets. Note that tRNAThr-derived tRFs (A) were exclusively found in SIV virions and that the scales of the y-axes differ in (B)-(F). (TIF) [file pone.0075063.s007.tif]
