# Supplementary material for: Mapping the Small RNA Content of Simian Immunodeficiency Virions (SIV)
Source: PLoS One. 2013 Sep 23;8(9):e75063. doi: 10.1371/journal.pone.0075063 (PMC3781035; doi:10.1371/journal.pone.0075063)
Supplement: Table S1 — Complete list of miRNAs identified in virion- and mock preparations. (PDF) [file pone.0075063.s008.pdf]

Table S1:
Complete list of miRNAs identified in virion- and mock preparations

miRNA	SIV	mock
hsa-let-7a-5p	631	371
hsa-let-7b-5p	89	82
hsa-let-7c	67	48
hsa-let-7d-5p	4	8
hsa-let-7e-5p	25	10
hsa-let-7f-5p	745	377
hsa-miR-15a-5p	6	4
hsa-miR-16-5p	7	4
hsa-miR-19a-3p	22	9
hsa-miR-19b-3p	70	56
hsa-miR-21-5p	14	11
hsa-miR-21-3p	1	1
hsa-miR-23a-3p	5	1
hsa-miR-24-3p	32	64
hsa-miR-25-3p	3	10
hsa-miR-26a-5p	127	109
hsa-miR-26b-5p	26	48
hsa-miR-27a-3p	1	1
hsa-miR-28-3p	1	1
hsa-miR-29a-5p	10	5
hsa-miR-29a-3p	138	88
hsa-miR-30a-5p	119	76
hsa-miR-30a-3p	3	3
hsa-miR-32-5p	1	1
hsa-miR-92a-3p	8	6
hsa-miR-93-5p	4	1
hsa-miR-96-5p	1	1
hsa-miR-98-5p	5	2
hsa-miR-99a-5p	100	79
hsa-miR-99a-3p	1	3
hsa-miR-100-5p	165	70
hsa-miR-101-3p	118	100
hsa-miR-29b-3p	7	3
hsa-miR-103a-3p	22	10
hsa-miR-199a-3p	7	18
hsa-miR-30c-5p	12	6
hsa-miR-30c-2-3p	1	1
hsa-miR-30d-5p	21	15
hsa-miR-7-5p	19	9
hsa-miR-10b-5p	74	2
hsa-miR-34a-5p	21	19
hsa-miR-181a-5p	45	10
hsa-miR-181b-5p	21	3
hsa-miR-181c-5p	1	2
hsa-miR-182-5p	26	31
hsa-miR-183-5p	16	25

hsa-miR-199b-5p	5	3		
hsa-miR-204-5p	2	3		
hsa-miR-217	2	2		
hsa-miR-218-5p	7	1		
hsa-miR-223-3p	1	2		
hsa-let-7g-5p	38	29		
hsa-let-7i-5p	116	36		
hsa-miR-1	112	12		
hsa-miR-23b-3p	7	4		
hsa-miR-27b-3p	8	12		
hsa-miR-30b-5p	4	6		
hsa-miR-124-3p	12	6		
hsa-miR-125b-5p	284	272		
hsa-miR-128	8	8		
hsa-miR-135a-5p	30	4		
hsa-miR-137	12	5		
hsa-miR-138-5p	9	12		
hsa-miR-140-3p	6	11		
hsa-miR-142-3p	21	32		
hsa-miR-143-3p	425	352		
hsa-miR-145-5p	2	3		
hsa-miR-152	10	14		
hsa-miR-163	1422	2365	Acc. no.	AJ535831.1
hsa-miR-9-5p	558	181		
hsa-miR-9-3p	46	15		
hsa-miR-125a-5p	3	3		
hsa-miR-126-5p	9	7		
hsa-miR-126-3p	35	40		
hsa-miR-127-3p	5	9		
hsa-miR-134	1	2		
hsa-miR-136-5p	12	5		
hsa-miR-146a-5p	3	14		
hsa-miR-149-5p	5	3		
hsa-miR-185-5p	19	15		
hsa-miR-186-5p	3	8		
hsa-miR-190a	4	2		
hsa-miR-195-5p	36	36		
hsa-miR-206	6	1		
hsa-miR-320a	6	10		
hsa-miR-29c-3p	102	78		
hsa-miR-219-2-3p	12	1		
hsa-miR-99b-5p	8	5		
hsa-miR-30e-5p	71	48		
hsa-miR-30e-3p	3	4		
hsa-miR-361-5p	3	2		
hsa-miR-369-3p	2	2		
hsa-miR-374a-5p	6	4		
hsa-miR-378a-3p	8	9		
hsa-miR-379-5p	5	5		
hsa-miR-340-5p	4	5		

hsa-miR-342-3p	7	2	
hsa-miR-151a-3p	5	4	
hsa-miR-148b-3p	1	1	
hsa-miR-338-3p	234	233	
hsa-miR-335-5p	6	1	
hsa-miR-423	547	453	Acc. no. NR_029945.1
hsa-miR-425-5p	6	3	
hsa-miR-451a	9	20	
hsa-miR-452-5p	3	3	
hsa-miR-486-5p	2	12	
hsa-miR-491-5p	2	1	
hsa-miR-146b-5p	4	4	
hsa-miR-497-5p	12	12	
hsa-miR-532-5p	13	7	
hsa-miR-92b-3p	3	1	
hsa-miR-574-3p	1	1	
hsa-miR-598	2	2	
hsa-miR-1224-5p	2	1	
hsa-miR-708-5p	1	3	
hsa-miR-1246	11	12	
hsa-miR-1249	1	1	
hsa-miR-103b	22	10	
hsa-miR-3065-5p	228	223	
hsa-miR-3676-5p	866	54	identical to $tRNA^{Thr}$ (Acc. no. X64276)
hsa-miR-4448	2	1	
hsa-miR-4488	2	3	
hsa-miR-4492	4	8	
hsa-miR-4508	2	6	
hsa-miR-4516	2	2	
hsa-miR-4532	12	3	
hsa-miR-3529-3p	13	5	
hsa-miR-6087	2	2	
**total**	**8387**	**6606**	
