# Supplementary material for: Mapping the Small RNA Content of Simian Immunodeficiency Virions (SIV)
Source: PLoS One. 2013 Sep 23;8(9):e75063. doi: 10.1371/journal.pone.0075063 (PMC3781035; doi:10.1371/journal.pone.0075063)
Supplement: Table S2 — Abundance of exosomal or virion-associated tRFs. (PDF) [file pone.0075063.s009.pdf]

Supplementary Table S2: Abundance of exosomal or virion-associated tRFs

	reads in SIV	reads in mock	ratio
**$tRNA^{Lys3}$**	107594	12531	8,59
**$tRNA^{Lys1,2}$**	10367	1443	7,18
**$tRNA^{Thr}$**	543	0	unique
**$tRNA^{Arg}$**	901	56	16,09
**$tRNA^{Met}$**	1206	125	9,65
**$tRNA^{Asn}$**	970	153	6,34
**$tRNA^{Tyr}$**	1786	342	5,22
**$tRNA^{Gln}$**	12764	3190	4,00
**$tRNA^{His}$**	23161	6328	3,66
**$tRNA^{Gly}$**	15683	13370	1,17
**$tRNA^{Glu}$**	13416	31003	0,43
**$tRNA^{Val}$**	4855	3686	1,32
**RNA^{Leu}**	3021	2231	1,35
**$tRNA^{Ala}$**	1152	763	1,51
**$tRNA^{Asp}$**	382	221	1,73
**$tRNA^{Ile}$**	77	156	0,49
